# Transcriptomic Response and Molecular Adaptation Mechanisms of Common Carp (*Cyprinus carpio*) Intestine Under Dual Stress of High Temperature and Zinc

**DOI:** 10.3390/ani16091334

**Published:** 2026-04-27

**Authors:** Xiaoying Jiang, Junli Zheng, Zilong Jiang, Yiyu Cao, Ying Jiang, Wei Hu, Deliang Li, Fan Yu

**Affiliations:** 1 College of Fisheries, Hunan Agricultural University, Changsha 410128, China; 2 State Key Laboratory of Developmental Biology of Freshwater Fish, Engineering Research Center of Polyploid Fish Reproduction and Breeding of the State Education Ministry, College of Life Sciences, Hunan Normal University, Changsha 410081, China; 3 State Key Laboratory of Breeding Biotechnology and Sustainable Aquaculture, Institute of Hydrobiology, Chinese Academy of Sciences, Wuhan 430072, China

**Keywords:** *Cyprinus carpio*, transcriptome, heat stress, zinc, intestinal tissue

## Abstract

This study employed a 2 × 2 factorial design to investigate the transcriptomic responses of common carp intestine under the single and combined stress of high temperature and zinc. Results from Illumina RNA-seq, WGCNA, and qPCR showed high-quality sequencing data with good reproducibility. High temperature was the core driver of global transcriptomic reprogramming, inducing numerous DEGs via activating metabolic and stress signaling pathways. Zinc alleviated heat stress by reducing DEGs by 43.2% and activating DNA repair and cell cycle pathways. WGCNA identified core modules for heat response and zinc mitigation, revealing “emergency defense” and “repair adaptation” strategies. This study enriches the teleost stress adaptation theory and provides a scientific basis for healthy carp aquaculture.

## Simple Summary

This study explored the intestinal transcriptomic responses of common carp to high-temperature and zinc dual stress via a 2 × 2 factorial design and RNA-seq. High temperature triggered massive transcriptomic reprogramming by activating stress and metabolic pathways, while zinc reduced heat-induced differential genes by 43.2% through promoting DNA repair and cell cycle regulation. WGCNA identified distinct modules for heat defense and zinc-mediated repair, offering insights for heat-tolerant carp breeding and precision zinc application in aquaculture.

## 1. Introduction

Aquaculture is one of the fastest-growing sectors of the global food industry, supplying approximately 50% of the animal protein consumed by humans worldwide, and this proportion is projected to rise to 53% by 2030 [1]. Over the past few decades, the aquaculture industry has achieved rapid and sustained development globally, particularly in China, which has become the world’s largest producer and consumer of aquatic products. However, the sustainable development of this sector is increasingly constrained by various environmental stressors, among which heat stress caused by global warming is the most prominent and widespread threat. The Earth’s climate is warming rapidly, with extreme high-temperature events becoming more frequent, intense, and prolonged, resulting in an elevated risk of heat stress in farmed fish across the globe [2]. Elevated water temperatures accelerate the proliferation of pathogenic microorganisms and reduce dissolved oxygen content. They also directly disrupt the physiological homeostasis of fish, ultimately leading to severe economic losses [3]. Fish are poikilothermic animals: their body temperature changes with the ambient water temperature. Most fish species have adapted to survive and grow within a specific optimal temperature range. Temperatures beyond this range can trigger growth retardation, developmental deformities, reduced disease resistance, and even mass mortality [4,5]. Therefore, investigating the response mechanisms of fish to temperature stress is critical for determining optimal rearing temperatures, elucidating thermal adaptation, supporting selective breeding of heat-tolerant varieties, guiding aquaculture site selection, managing water temperature in intensive systems, and improving overall sustainability [6,7,8,9].

The response of fish to heat stress is a highly complex and multi-level biological process. At individual and behavioral levels, heat stress leads to abnormal behaviors such as reduced feed intake in Amur sturgeon (*Acipenser schrenckii*) and Atlantic salmon (*Salmo salar*), as well as disordered respiratory rhythm and irregular swimming behavior in common carp (*Cyprinus carpio communis*) [10,11,12]. At physiological and biochemical levels, heat stress alters energy metabolism, oxidative stress responses, antioxidant system activity, immune function, and neuroendocrine regulation. Previous studies have shown that heat stress up-regulates the activities of key glycolytic enzymes, catalase, and other antioxidant enzymes in olive flounder (*Paralichthys olivaceus*), turbot (*Scophthalmus maximus*), and Yangtze sturgeon (*Acipenser dabryanus*) [13,14]. It also inhibits the phagocytic activity of immune cells and lysozyme activity in Nile tilapia (*Oreochromis niloticus*) and disrupts the secretion of reproductive and thyroid hormones in several teleost species [6,15,16].

At the molecular level, the expression of heat shock proteins (HSPs), which play a core role in maintaining intracellular protein homeostasis, is significantly increased under heat stress in various fish species. HSPs protect cells from thermal damage by promoting the correct folding of nascent peptides and the refolding of denatured proteins [17,18]. In recent years, RNA sequencing (RNA-Seq) transcriptomic technology has been widely applied to decipher the complex molecular mechanisms of fish in response to heat stress [19,20]. Existing studies have shown that heat stress up-regulates genes related to protein folding and degradation, oxidative stress, immune response, and cell signal transduction in the gill tissue of *Chinook salmon* [21]. It up-regulates genes associated with HSPs, fatty acid metabolism, and apoptosis in the liver and brain of grass carp [22]. In the liver of Yangtze sturgeon, heat stress affects genes involved in HSPs, ubiquitination, and immune pathways [14]. These findings reveal that fish activate specific biological processes and physiological functions to adapt to high temperatures and reduce damage. However, most existing studies on fish heat stress have focused on metabolic organs such as the liver [23,24,25]. The intestine, a key organ for nutrient absorption, immune defense, and environmental interaction, has received much less attention, especially under combined environmental stress.

Zinc is an essential trace element for fish growth and development. It serves as a cofactor for more than 300 metalloenzymes and a structural component of zinc finger domains in numerous transcription factors. It participates in a wide spectrum of biological processes, including reactive oxygen species scavenging, DNA damage repair, protein folding and degradation, and transcriptional regulation of immune- and stress-related genes [26,27]. The biological effects of zinc exhibit a distinct concentration-dependent biphasic characteristic. At nutritional concentrations, zinc promotes growth, antioxidant capacity, and immune function [28,29]. However, when the environmental zinc concentration exceeds the tolerance threshold, it becomes toxic, inducing oxidative stress, mitochondrial dysfunction, cellular apoptosis, and disruption of normal physiological processes [30,31,32,33,34]. To date, existing studies on the anti-stress role of zinc in aquaculture have largely focused on dietary supplementation [35,36]. The regulatory role of waterborne sublethal zinc exposure under heat stress—especially its interaction with high temperature at the transcriptomic level in the fish intestine—remains completely unexplored. In addition, transcriptomic changes under combined stress are far more complex than under single stress. The alteration in the number of differentially expressed genes cannot be simply equated with the degree of stress damage or protection, which requires systematic experimental design and multi-dimensional bioinformatics analysis.

Common carp is a dominant freshwater farmed species in China, with advantages of fast growth rate, wide environmental adaptability, strong stress resistance, and low farming cost [37]. Its thermal tolerance range is 3–35 °C [38]. Although the upper limit of its optimal growth range under normal aquaculture conditions is approximately 35 °C, temperatures above this threshold (e.g., 38 °C) have been widely used as experimental heat stress conditions and have been shown to induce significant transcriptomic and physiological responses without causing acute mortality when a gradual heating protocol is applied [39,40,41,42]. In actual aquaculture production, sustained high water temperatures in summer often cause severe physiological disorders, reduced growth performance, and increased disease incidence, leading to huge economic losses [43,44,45]. The intestine is the main site of nutrient digestion and absorption, the first line of immune defense against exogenous pathogens and toxic substances, and the core interface for interaction between the organism and the external aquatic environment. It is one of the most sensitive target organs to environmental stressors, including high temperature [46]. However, the transcriptomic characteristics and underlying molecular regulatory mechanisms of the carp intestine in response to high-temperature stress have not yet been systematically elucidated.

In this study, we employed a 2 × 2 factorial experimental design, including four treatment groups: control group, high-temperature stress group, waterborne sublethal zinc exposure group, and combined high-temperature and sublethal zinc exposure group. The aim of this approach was to systematically investigate the transcriptomic profiles of common carp intestine under the single and combined stress of high temperature and sublethal zinc. Using Illumina high-throughput RNA sequencing technology, we performed differential expression gene analysis, Gene Ontology (GO) and Kyoto Encyclopedia of Genes and Genomes (KEGG) functional enrichment analysis, and weighted gene co-expression network analysis (WGCNA) to decode the transcriptomic response characteristics of common carp intestine to high-temperature stress and to clarify the interactive effect between sublethal zinc exposure and high-temperature stress. Meanwhile, quantitative real-time polymerase chain reaction (qRT-PCR) was applied to verify the reliability of the transcriptome sequencing data and to screen the key functional pathways, core co-expression modules and hub genes that mediate the high-temperature response of carp intestine and the regulatory effect of zinc. The findings of this research will not only deepen the understanding of the molecular mechanism underlying the thermal stress response in common carp and elaborate the dual regulatory role of trace element zinc in the combined environmental stress response of fish, but also provide candidate molecular targets for the genetic breeding of heat-tolerant common carp strains, offer a theoretical basis for the scientific application of zinc in the healthy culture of common carp during high-temperature seasons, and serve as an important reference for the study of combined environmental stress response mechanisms in other teleost species.

## 2. Materials and Methods

### 2.1. Stress Experiment and Sample Collection

Healthy 4-month-old common carp used in the experiment were bred by the Institute of Hydrobiology, Chinese Academy of Sciences, with an initial average body length of (6.55 ± 0.72) cm and an average body weight of (6.06 ± 1.65) g. They were acclimated in glass aquariums (60 cm × 30 cm × 40 cm) for 7 days at a stocking density of 20 fish per aquarium. During acclimation, the culture environment was strictly controlled as follows: water temperature (15.0 ± 0.5) °C (set temperature for the normal temperature control group), pH (7.5 ± 0.2), dissolved oxygen content ≥8 mg/L, and photoperiod of 12 h light/12 h dark (light intensity: 3000–5000 lx). Commercial compound feed produced by Qingdao Qihao Nutrition Technology Co., Ltd. (Qingdao, China, crude protein content 47.89%, crude fat content 15.69%, crude fiber ≤ 5%, and crude ash ≤ 18%) was fed at 08:00 and 18:00 daily, with the feeding amount accounting for 1% of the fish body weight. Residual bait and feces were promptly removed 2 h after feeding, and 30% of the culture water was replaced daily to maintain water quality stability. Feeding was stopped for all groups of fish 24 h before the end of acclimation to reduce the interference of digestive physiological activities on the experimental results. During the period, water quality indicators such as ammonia nitrogen (≤0.05 mg/L) and nitrite (≤0.1 mg/L) were monitored daily to ensure no pathogenic contamination or environmental stress.

A 2 × 2 fully randomized factorial design was adopted in this experiment, with the normal temperature set at 15 °C, high temperature at 38.5 °C to induce heat stress exposure, and metal zinc addition concentration at 15 μM (a sublethal concentration corresponding to ~50% of the 96 h-LC_50_) [47]. A total of 4 treatment groups were established. High-temperature stress was induced by gradient heating: the water temperature was first stabilized at 24 °C, then heated to the target temperature of 38.5 °C at a rate of 4 °C/h, and stabilized for 24 h as a heat acclimation period before the official 21-day experiment to avoid acute temperature-shock-induced stress damage to the fish. The high-zinc treatment was achieved by adding analytical-grade ZnCl_2_ (Sinopharm Chemical Reagent Co., Ltd., Shanghai, China, purity ≥99.0%), prepared fresh before use. The experimental period was 21 days, during which the culture conditions were consistent with those during acclimation. The activity status, feeding situation, and mortality of the fish were observed and recorded daily, and abnormal individuals were promptly isolated and recorded. Each treatment consisted of three independent tanks, and one fish was sampled from each tank for RNA-seq, resulting in three biological replicates per treatment (Figure S7). At the end of the experiment, a total of 12 samples were collected in the four treatment groups, anesthetized with 200 mg/L MS-222 (tricaine methanesulfonate, Sigma-Aldrich, St. Louis, MO, USA, buffered with NaHCO_3_ before use to adjust the solution pH to 7.0–7.5) until loss of voluntary movement and slow gill cover breathing. Midgut tissues (the middle intestinal segment, with contents removed) were rapidly dissected in a sterile ultra-clean workbench, rinsed 3 times with pre-cooled sterile normal saline to remove surface blood and impurities, blotted dry with filter paper, and quickly aliquoted into RNase-free centrifuge tubes (Eppendorf, Hamburg, Germany), with approximately 50 mg of tissue per tube. Samples were snap-frozen in liquid nitrogen for 60 min and immediately transferred to a −80 °C ultra-low temperature refrigerator (Thermo Fisher Scientific, Waltham, MA, USA) for subsequent total RNA extraction. The entire sample collection process was strictly completed within 1 h, and all instruments in contact with samples were treated with RNase inactivation to avoid RNA degradation. Sample identification and submission names of each group corresponded strictly to ensure the traceability of subsequent experiments.

### 2.2. RNA Extraction, Library Construction and Sequencing

Total RNA was extracted from carp intestinal tissue samples of each treatment group using TRIzol reagent (Invitrogen, Carlsbad, CA, USA) according to the manufacturer’s instructions. RNA concentration and purity were detected using a Nanodrop 2000 spectrophotometer (Thermo Scientific, Waltham, MA, USA) to ensure the A_260_/A_280_ ratio was between 1.8 and 2.0. Meanwhile, RNA integrity was detected using an Agilent 2100 Bioanalyzer combined with an RNA 6000 Nano Kit (Agilent Technologies Inc., Santa Clara, CA, USA, 5067-1511), and samples with RIN ≥ 7.0 and total amount ≥ 1 μg were used for subsequent experiments. Library construction was performed using a NEBNext Ultra II RNA Library Prep Kit for Illumina (New England Biolabs Inc., Ipswich, MA, USA). Briefly, mRNA with poly(A) tails was enriched using oligo (dT) magnetic beads then randomly fragmented by divalent cation-induced fragmentation. Fragmented mRNA was used as a template to synthesize cDNA with random oligonucleotides as primers. After purification of double-stranded cDNA, end repair and 3′ end A-tailing were performed, followed by adapter ligation. cDNA fragments of 400~500 bp were selected using AMPure XP beads, and the final library was obtained after PCR amplification and re-purification. Library quality was detected using an Agilent 2100 Bioanalyzer combined with an Agilent High Sensitivity DNA Kit (Agilent Technologies Inc., Santa Clara, CA, USA, 5067-4626). Total concentration was determined using a Quantifluor-ST fluorometer (Promega, Madison, WI, USA, E6090) combined with a Quant-iT PicoGreen dsDNA Assay Kit (Invitrogen, Carlsbad, CA, USA, P7589), and effective library concentration was quantitatively verified using StepOnePlus Real-Time PCR Systems (Thermo Scientific, Waltham, MA, USA). Multiple sample DNA libraries were normalized, mixed in equal volumes, serially diluted and quantified, then sequenced on an Illumina sequencing platform in paired-end (PE150) mode to generate raw sequencing data (raw data).

### 2.3. Sequencing Quality Control and Reference Genome Alignment

Raw data obtained from Illumina sequencing were quality-controlled using fastp software (v0.22.0) [48] to remove sequences with adapters at the 3′ end, reads with an average quality score below Q20, and sequences with a high proportion of unknown nucleotides (N), resulting in high-quality clean data (clean data). The reliability of the data was verified by counting the Q20, Q30, GC content, and error rate of clean reads, requiring Q20 ≥ 90% and Q30 ≥ 85%. The reference genome and gene model annotation files of common carp were downloaded from public genome databases (GCA_000951615.2, https://www.ncbi.nlm.nih.gov/assembly/GCA_000951615.2/, accessed on 13 May 2025). The gene annotation data were retrieved from the Ensembl database (release 100.2) (https://asia.ensembl.org/Cyprinus_carpio_carpio/Info/Index?, accessed on 10 June 2025). A reference genome index was constructed using HISAT2 software (v2.1.0) [49], and the quality-controlled clean reads were aligned to the reference genome in paired-end mode based on this index. HISAT2 can generate a splice junction database using gene model annotation files to improve alignment accuracy. After alignment, the proportions of Unique Mapped Reads, Multiple Mapped Reads, and the total mapping rate of each sample were counted to ensure a total mapping rate ≥ 80% to guarantee the reliability of subsequent analyses.

### 2.4. Differential Expression Gene (DEG) Analysis

Read Count values mapped to each gene were counted as raw expression levels using HTSeq software (v0.9.1) [50]. To eliminate the influence of gene length and sequencing depth on expression levels, normalization was performed using the FPKM (Fragments Per Kilobase of transcript per Million mapped reads) method [51]. For PE150 sequencing data, FPKM only counts the number of fragments where both reads can be aligned to the same transcript to accurately reflect the relative gene expression level. Differential expression analysis between groups was performed using DESeq software (v1.39.0) [52], with screening criteria set as |log_2_(Fold Change)| > 1 and adjusted significance (*p*.adjust) < 0.05 to obtain significantly differentially expressed genes. Subsequent GO functional enrichment analysis and KEGG pathway enrichment analysis by clusterProfiler (4.6.0) [53] were performed based on these DEGs to reveal the key molecular response mechanisms of carp intestinal tissues under high-temperature stress and zinc regulation.

### 2.5. WGCNA Analysis

WGCNA considers the strength and weight of relationships (correlation values) between nodes (genes) and uses the Topological Overlap Matrix (TOM) to measure gene–gene correlations, thereby identifying highly co-expressed gene sets. For the carp transcriptome dataset, we used the WGCNA package (1.72-5) to perform optimal soft threshold screening, gene clustering and module identification, and module–trait correlation analysis [54] and finally selected the gene modules most positively and negatively correlated with phenotypes for subsequent analysis. Because the subsequent functional enrichment analyses were performed at the module level using all genes within the selected modules, no additional hub gene filtering was applied. The R package clusterProfiler (4.6.2) was used for Gene Ontology (GO) and Kyoto Encyclopedia of Genes and Genomes (KEGG) functional annotation and enrichment analysis of genes. A *p*-adjust < 0.05 was considered significantly enriched.

### 2.6. qPCR Validation of Gene Expression

To confirm the reliability of transcriptome data, 9 DEGs were randomly selected for qRT-PCR validation [55]. Total RNA was extracted from intestinal samples of C, Zn, H, and H + Zn groups using TRIzol Reagent, and RNA concentration and integrity were detected using a NanoDrop spectrophotometer (Thermo Fisher Scientific, Waltham, MA, USA) and agarose gel electrophoresis. Reverse transcription was performed by incubating 1 μg RNA with 2.5 μM oligo dT primer, 1 mM deoxynucleotide triphosphate mixture, 20 U RNase inhibitor, and 100 U ReverTra Ace (Toyobo Life Science, Shanghai, China) in the appropriate buffer for 60 min at 42 °C and 5 min at 99 °C. Quantitative PCR was performed on a Bio-Rad CFX96 Real-Time PCR System (Bio-Rad, Hercules, CA, USA) using SYBR Green Realtime PCR Master Mix (Toyobo Life Science, Shanghai, China). The reaction conditions were 95 °C for 3 min, followed by 40 cycles at: 95 °C for 15 s, 60 °C for 15 s, and 72 °C for 15 s. The primers used for amplification are listed in Table S1. The expression values of the target genes were normalized to the amount of *β-actin* mRNA. Data analysis was performed using the 2^−ΔΔCT^ method [56]. Each experiment was performed in triplicate. These candidate genes were chosen for comparative analysis between qRT-PCR and transcriptome sequencing in order to verify the reliability of the transcriptomic data [3,57].

## 3. Results

### 3.1. Sequencing Data Quality Assessment and Gene Expression Quantification

Throughout the 21-day stress experiment, no mortality was recorded in any treatment group, with a 0% mortality rate in all common carp, ensuring the validity of subsequent sequencing and transcriptomic analysis. A total of 532,980,234 raw reads and 80,480,015,334 bp raw data were obtained from the transcriptome sequencing of 12 samples in the four treatment groups (Table 1). After removing low-quality data, a total of 520,706,000 clean reads and 78,292,946,876 bp clean data were obtained. Clean reads of C, H, Zn and H + Zn groups were 43,837,856–48,747,934, 39,549,320–41,610,548, 41,357,444–43,889,000 and 36,728,126–55,098,142, respectively; the clean data of these four groups were 6,591,368,894–7,307,488,228, 5,954,748,335–6,268,797,214, 6,219,696,666–6,590,457,092 and 5,527,373,339–8,292,685,814 bp, respectively. The Q20, Q30, and GC content of each sample were 97.92–98.93%, 94.39–97.08%, and 41.81–45.43%, respectively, which suggests that the sequencing quality is good.

The high-quality clean reads aligned with the reference genome showed that the total mapped rate of all samples ranged from 79.39% to 83.90% with an average of 81.70%, and the uniquely mapped rate ranged from 87.16% to 92.87% with an average of 90.10% (Table 2). Among the 12 samples, eight had a total mapped rate higher than 81%, and six had a uniquely mapped rate higher than 91%, which indicates that the transcriptome sequencing data have a good alignment result with the reference genome. The results of sequencing, quality control, and genome alignment showed that the transcriptome sequencing data were reliable, which could ensure the accuracy of subsequent analysis.

### 3.2. Differential Gene Expression Analysis

**Figure 1:**
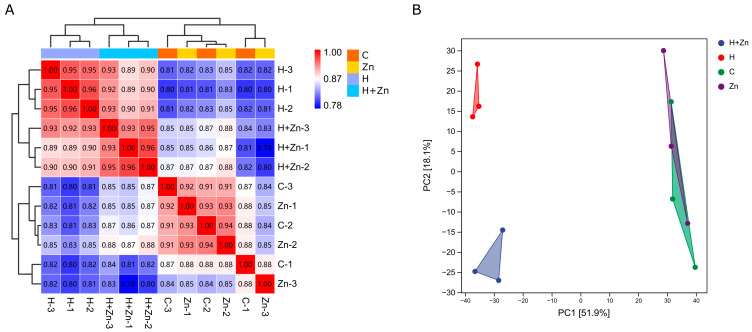
Transcriptomic profile clustering and principal component analysis (PCA) of common carp intestinal samples under different treatments. (**A**) Heatmap of Pearson correlation coefficients between biological replicates, with hierarchical clustering showing high within-group reproducibility. (**B**) PCA plot of the first two principal components.

**Figure 2:**
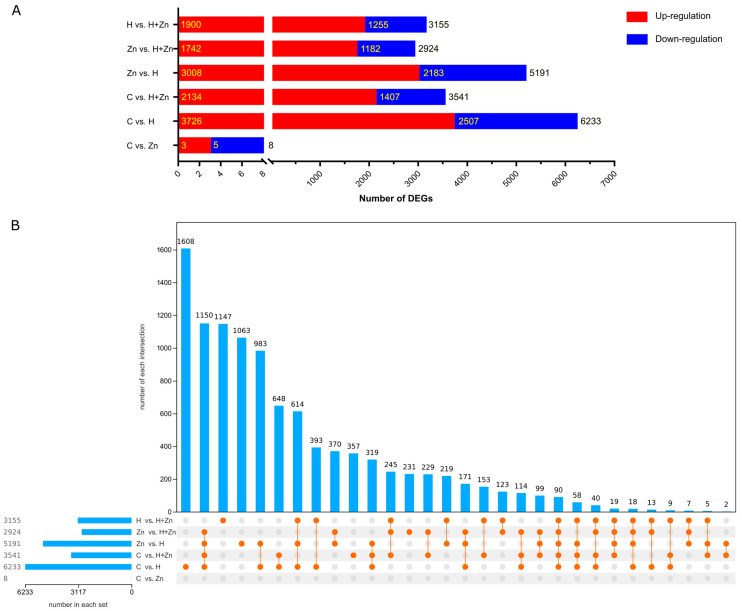
Analysis of differentially expressed genes (DEGs) in common carp intestine under different treatments. (**A**) Number of up-regulated (red) and down-regulated (blue) genes in each comparison group. (**B**) UpSet plot analysis showing the overlap of DEGs among different treatment comparisons. The left horizontal bar chart displays the total number of DEGs identified in each comparison. The matrix of dots in the middle indicates which comparisons are included in each intersection, with orange dots representing included comparisons and gray dots representing excluded ones. The top vertical bar chart shows the number of DEGs in each corresponding intersection.

**Figure 3:**
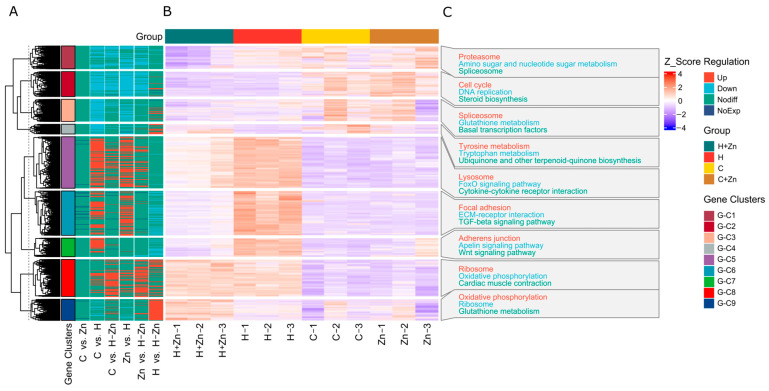
Hierarchical clustering and functional enrichment of differentially expressed genes (DEGs) in common carp intestine. (**A**) Hierarchical clustering heatmap of DEGs, with samples grouped by treatment and genes divided into 9 distinct clusters (G-C1 to G-C9) based on expression patterns. (**B**) Detailed view of the clustering heatmap showing the expression profiles of DEGs across all samples. (**C**) KEGG pathway enrichment analysis of genes in each cluster revealing the key biological processes and pathways associated with each expression pattern under different stress conditions.

As shown in Figure 1A, the Pearson correlation coefficients between parallel samples within the C (control), H (high-temperature), Zn (zinc-added), and H + Zn (high-temperature + zinc-added) groups were 0.87–0.91, 0.95–0.96, 0.85–0.93, and 0.93–0.96, respectively, indicating good reproducibility between biological replicates. Principal component analysis (PCA) showed that the first two principal components accounted for 70.0% of the total variance of the dataset (Figure 1B). Samples from the control group and high-zinc group were clustered closely, while the high-temperature group and high-temperature–high-zinc combined group were completely separated from the normal temperature groups along the PC1 axis, which demonstrated that high-temperature stress was the main driver of the overall expression profile differences in common carp, and the biological replicates within each treatment were well clustered with high experimental repeatability. Based on FPKM values, differentially expressed genes (DEGs) between different treatment groups were screened. The results (Figure 2A, Figure S1 and Table S2) showed that a total of eight (three up-regulated and five down-regulated), 6233 (3726 up-regulated and 2507 down-regulated), 3541 (2134 up-regulated and 1407 down-regulated), 5191 (3008 up-regulated and 2183 down-regulated), 2924 (1742 up-regulated and 1182 down-regulated), and 3155 (1900 up-regulated and 1255 down-regulated) DEGs were detected in the C vs. Zn, C vs. H, C vs. H + Zn, Zn vs. H, Zn vs. H + Zn, and H vs. H + Zn comparison groups, respectively. Notably, the number of DEGs was 6233 in the H group and 3541 in the H + Zn group, with a 43.2% reduction in the latter group. The number of DEGs increased sharply in the high-temperature treatment group (C vs. H) compared with the control vs. zinc group (C vs. Zn), while the DEG number in the H + Zn group (C vs. H + Zn) was lower than that in the H group alone, implying that zinc supplementation may mitigate the gene expression alterations induced by high-temperature stress (Figure S1 and Figure 3A). A total of 10505 DEGs were identified across the six comparison groups (Table S3). UpSet plot analysis (Figure 2B) identified 648 DEGs shared between the single high-temperature stress (C vs. H) and high-temperature plus zinc combined stress (C vs. H_Zn) comparisons. These DEGs were only significantly differentially expressed in high-temperature-related comparisons and were not affected by zinc exposure. A total of 90 DEGs were shared by all five comparisons except C vs. Zn. Furthermore, the six comparisons contained 4406 group-specific DEGs in total.

The clustering results of differentially expressed genes (DEGs) showed that these DEGs could be divided into nine independent gene clusters (G-C1 to G-C9) (Figure 3A and Figure S2). Each cluster exhibited a specific expression pattern across the four treatment groups, with significant changes observed in the high-temperature-treatment-related groups (Figure 3B). Gene clusters with prominent expression changes in the high-temperature treatment group were mainly enriched in pathways related to stress response and cellular homeostasis maintenance (Figure 3C), such as proteasome, cell cycle, and DNA replication. In contrast, gene clusters with alleviated regulatory trends in the H + Zn group were mostly associated with stress-adaptation-related pathways, including the lysosome and FoxO signaling pathway.

### 3.3. GO Annotation Results of DEGs in Carp Intestinal Tissues

**Figure 4:**
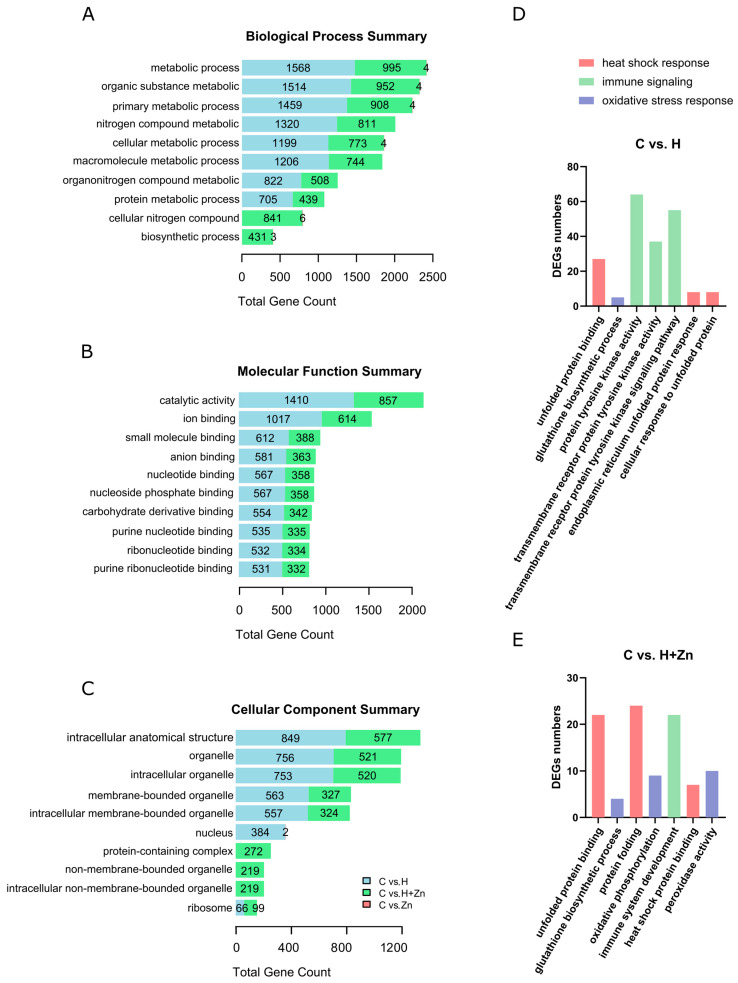
Gene Ontology (GO) classification of differentially expressed genes (DEGs) in common carp under different temperature and zinc stress treatments. (**A**) Biological Process GO terms enriched. (**B**) Molecular Function GO terms. (**C**) Cellular Component GO terms. (**D**,**E**) GO enrichment analysis of differentially expressed genes related to heat shock response, oxidative stress, and immune signaling in the comparisons C vs. H and C vs. H + Zn. The x-axis shows specific GO terms. The y-axis shows the number of DEGs.

As shown in Figure 4 and Figure S3, the Gene Ontology (GO) annotation results of differentially expressed genes (DEGs) in carp intestinal tissues under four treatments (control (C), high temperature (H), zinc added (Zn), and high temperature + zinc added (H + Zn)) covered three major functional categories: Biological Process (BP), Molecular Function (MF), and Cellular Component (CC). Overall, the total number of DEGs in the three GO categories of high-temperature-related treatment groups (H, H + Zn) was significantly higher than that of the control group and the zinc-only (Zn) group. Among them, the proportion of DEGs related to catalytic activity in the H group reached 20.42%, and the number of DEGs in the H group in BP, MF, and CC was 9793, 6906, and 3928, respectively, while the corresponding numbers in the H + Zn group were 7402, 4321, and 2584. In contrast, the number of DEGs in the Zn group (BP: 29, MF: 0, CC: 2) and the C group (BP: 0, MF: 0, CC: 0) was at a low level, indicating that high-temperature stress is the main factor inducing DEG enrichment in carp intestines, and zinc addition has a certain regulatory effect on the total number of DEGs induced by high temperature.

To further clarify the specific biological pathways influenced by high temperature and zinc addition, we screened GO terms directly associated with heat shock response, oxidative stress, and immune signaling from the GO enrichment results (Table S4). Figure 4D,E show that among the heat-shock-response-related GO terms, unfolded protein binding (GO: 0051082) was significantly enriched in both the H and H + Zn groups, with higher enrichment significance in the H + Zn group. However, endoplasmic reticulum unfolded protein response (GO: 0030968) was enriched only in the H group, whereas protein folding (GO: 0006457) and heat shock protein binding (GO: 0031072) were enriched exclusively in the H + Zn group. Regarding oxidative-stress-related GO terms, glutathione biosynthetic process (GO: 0006750) was enriched in both groups, but peroxidase activity (GO: 0004601) and oxidative phosphorylation (GO: 0006119) were enriched only in the H + Zn group. For immune-related GO terms, protein tyrosine kinase activity (GO: 0004713) and its signaling pathway were highly enriched only in the H group. In contrast, immune system development (GO: 0002520) was enriched exclusively in the H + Zn group, and no enrichment of protein tyrosine kinase-related pathways was detected in this group.

### 3.4. WGCNA Results

**Figure 5:**
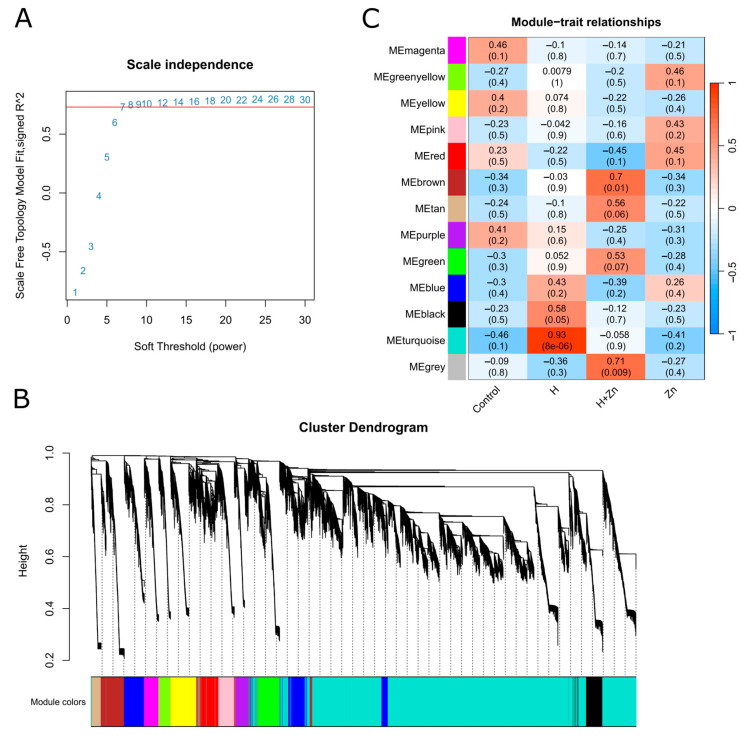
Weighted gene co-expression network analysis (WGCNA) of common carp intestinal genes under dual stress. (**A**) Soft threshold determination. Red line indicates the chosen soft threshold power = 7, at which signed R^2^ = 0.731, meeting the scale-free topology assumption. (**B**) Gene clustering dendrogram and module assignment. (**C**) Module–trait relationship heatmap.

Weighted gene co-expression network analysis (WGCNA) was used to integrate the dataset. As shown in Figure 5A, when the power was 7, the signed R^2^ reached 0.731, indicating that the network already conforms to a scale-free distribution. Therefore, the soft threshold power = 7 was selected as the optimal soft threshold for subsequent analysis, which met the requirements for constructing a weighted gene co-expression network. Subsequent clustering analysis showed that genes in the same branch were divided into the same module (Figure 5B). Co-expression module construction and trait correlation analysis were performed on the transcriptome data. Based on the similarity of gene expression profiles, all genes were clustered into 13 independent co-expression modules (Figure 5C), among which the turquoise module contained the largest number of genes, being the core module with the largest scale in the intestinal gene co-expression network.

As the module–trait correlation analysis showed (Figure 5C and Table S5), for the high-temperature trait (H), the most positively correlated module was the turquoise module (22,974 genes; *r* = 0.93, *p* = 8.6 × 10^−6^), and the grey module (20 genes; *r* = −0.36, *p* = 0.3) showed the most negative trend; for the zinc-only trait (Zn), the green-yellow module (864 genes; *r* = 0.46, *p* = 0.1) showed the most positive trend and the turquoise module (*r* = −0.41, *p* = 0.2) showed the most negative trend. For the combined high-temperature + zinc trait (H + Zn), the most positively correlated module was the grey module (*r* = 0.71, *p* = 0.009), and the red module (1487 genes; *r* = −0.45, *p* = 0.1) showed the most negative trend. These module-level gene sets served as the screening threshold for all downstream functional enrichment analyses, providing an important co-expression network framework for subsequent analysis of key genes and regulatory pathways.

### 3.5. Functional Enrichment Characteristics of WGCNA-DE Common Genes

**Figure 6:**
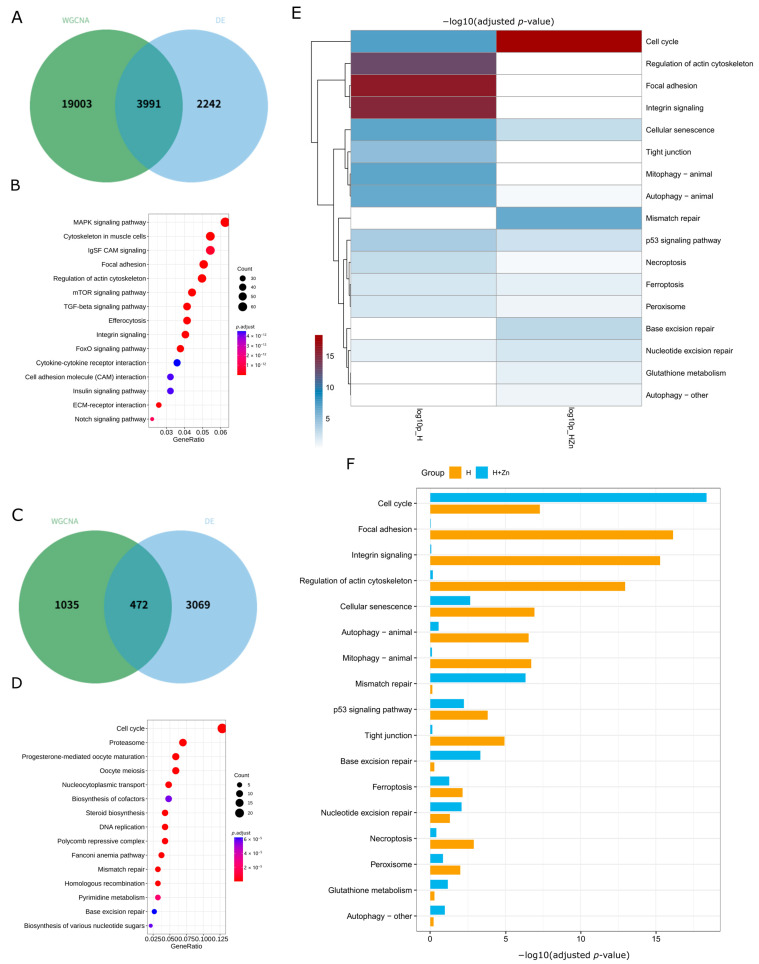
Functional enrichment analysis of WGCNA core modules in common carp intestine under H and H + Zn combined stress. (**A**,**C**) Venn diagrams of WGCNA module genes and DEGs: (**A**) in C vs. H comparison; (**C**) in C vs. H + Zn comparison. (**B**,**D**) KEGG pathway enrichment scatter plots for overlapping DEGs in (**A**) and (**C**), respectively. Heatmap (**E**) and bar plot (**F**) showing the core functional pathways among the 20 commonly enriched KEGG pathways (*p*.adjusted < 0.05) in C vs. H and C vs. H + Zn comparisons; the full list of 20 pathways is provided in Table S6.

Based on the results of the gene module and trait correlation analysis, the gene modules most positively and negatively correlated with each phenotype were selected and intersected with DEGs (Figure 6A,C and Figure S4). Subsequently, functional annotation was performed on the common genes screened by WGCNA and DE analysis. The results showed that the functional enrichment of these common genes was significantly treatment-dependent, mainly concentrating on three major GO categories (Biological Process (BP), Molecular Function (MF), and Cellular Component (CC)) (Figure S5) and multiple key KEGG signaling pathways (Figure 6B,D and Figure S6), providing core targets for analyzing the molecular regulatory network of intestinal responses to different environmental stresses. From the perspective of gene number characteristics, the scale of common genes in the high-temperature single-stress group was the largest (3991), that in the high-temperature + high-zinc double-stress group was 472, and that in the high-zinc single-stress group was 6 (Figure S4), without large-scale significant common gene enrichment. This indicates that high temperature is the main stress factor driving the coordinated response of intestinal gene co-expression and differential expression, and zinc addition can significantly change the number and functional direction of common genes.

Under high-temperature single stress, intestinal common genes were significantly enriched in the cytoskeleton, focal adhesion, FoxO, MAPK, mTOR, and TGF-beta signaling pathways (Figure 6B). In the CC category, cellular anatomical entities, protein complexes, and cell junctions dominated, with non-membrane-bound organelles, plasma membrane, and adherens junctions showing a *p*-adjust < 0.03, indicating regulation of cellular structure homeostasis and transmembrane signal transduction (Figure S5A). In high-temperature + high-zinc double stress, core pathways shifted to cell cycle, DNA replication, mismatch repair, and proteasome (Figure 6D). BP terms like chromosome segregation had a *p*-adjust as low as 1 × 10^−6^; MF focused on ATPase activity and CC enriched proteasome complexes and microtubule cytoskeletons, reflecting the alleviation of stress via cell proliferation, DNA repair, and protein degradation (Figure S5B). High-zinc single stress only weakly enriched a few metabolism-related GO terms with a small gene scale (Figure 4A–C), exerting limited effects on intestinal gene expression. In summary, functional differentiation was significant: high temperature alone drove cellular structure and signal-transduction-related genes, double stress favored cell cycle and DNA repair genes, and single zinc stress had minimal impact.

To identify biological processes involved in the intestinal response to high temperature and the potential protective role of zinc, we performed KEGG enrichment analysis on differentially expressed genes (Figure 6). A total of 20 pathways were commonly enriched in both H and H + Zn groups (Table S6). The heatmap (Figure 6E) and bar plot (Figure 6F) visualizes the −log10(*p.*adjusted) of all commonly enriched KEGG pathways in the H and H + Zn groups. Barrier-related pathways including focal adhesion, integrin signaling, regulation of actin cytoskeleton, and tight junction showed high −log10(*p*.adjusted) in the H (4.93–16.14) but dropped to near zero (0.03–0.17) in the H + Zn group. Cell death and autophagy pathways (mitophagy, autophagy animal, necroptosis, and ferroptosis) followed a similar pattern: −log10(*p*.adjusted) ranged from 2.15 to 6.71 in H and decreased to 0.11–1.26 in H + Zn group. In contrast, DNA repair pathways (mismatch repair, base excision repair, and nucleotide excision repair) had low −log10(*p*.adjusted) in H (0.14–1.30) but were markedly higher in the H + Zn group (2.08–6.34). The cell cycle pathway was highly enriched in both groups, with a −log10(*p*.adjusted) of 7.29 in the H and 18.37 in the H + Zn group. Oxidative-stress-related pathways (peroxisome and glutathione metabolism) showed modest differences between the two groups. p53 signaling and cellular senescence had a −log10(*p*.adjusted) of 3.82–6.93 in the H and 2.24–2.66 in the H + Zn group. Although immune-related pathways (e.g., cytokine–cytokine receptor interaction) were not commonly enriched, they were significantly enriched in the H group alone (Table S6).

### 3.6. Validation of Gene Expression Pattern by qPCR

**Figure 7:**
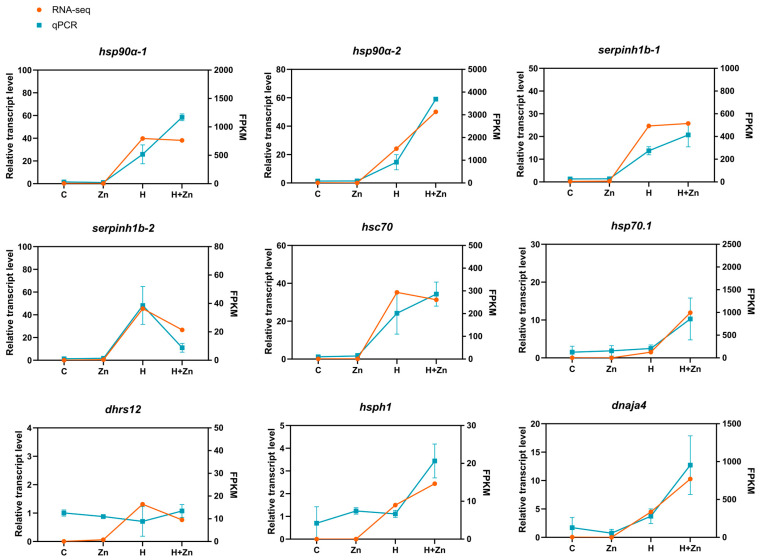
Comparison of expression patterns between qPCR and RNA-Seq for nine stress-responsive genes in the intestine of common carp under different treatments.

To validate the differentially expressed genes (DEGs) identified by RNA-seq, nine representative genes were selected for quantitative real-time PCR (qPCR) verification. These candidates included key regulators of cellular stress responses (HSP70.1, an ATP-dependent HSP70 family chaperone; HSPH1; and DNAJA4, an HSP70 co-chaperone), a critical component of collagen synthesis and extracellular matrix (ECM)–receptor interactions (SERPINH1B), and a zinc finger protein (ZFP36L1). The qPCR analysis results (Figure 7) revealed a high concordance in the expression patterns of all selected genes with the RNA-seq data, thereby confirming the reliability of the DEG profiling results.

## 4. Discussion

### 4.1. Intestinal Transcriptomic Reprogramming Under Heat Stress

DEG analysis showed that high temperature induced massive transcriptomic reprogramming in the common carp intestine (6233 DEGs in C vs. H versus only eight DEGs in C vs. Zn), representing a nearly 800-fold increase in DEG number compared with the C vs. Zn group (Figure S1). This indicates that heat stress is the primary driver of global gene expression remodeling, consistent with transcriptomic studies in other fish species [39,58]. GO enrichment showed that DEGs in the H group were mainly enriched in organic substance metabolism, carbohydrate metabolism and cellular stress response, with catalytic-activity-related DEGs accounting for 20.42%. These findings suggest that intestinal epithelial cells may rely on metabolic reprogramming to ensure energy supply for nutrient absorption and immune defense. This metabolic prioritization appears to be a primary adaptive strategy of the intestine [59]. Similar metabolic adjustments have been reported in rainbow trout and zebrafish [58,60].

The intestinal barrier of fish is particularly vulnerable to heat stress [58]. The tight junction pathway was significantly enriched in the H group (−log10(*p*.adjust) = 4.93) but dropped to near zero in the H + Zn group (0.14), suggesting that zinc is associated with modulated transcriptional changes related to intestinal barrier integrity under heat stress (Table S6). Tight junctions, composed of claudins, occludin and ZO proteins, are essential for intestinal integrity and paracellular permeability [58]. In grass carp, intestinal injury down-regulates *claudin-3*, *claudin-12*, *claudin-b*, *claudin-c*, *claudin-15a*, *ZO-3* and *occluding* [61]. In rainbow trout, heat stress similarly down-regulates *ZO-1*, *occludin* and *claudin* [62]. The reduced tight junction enrichment in the H + Zn group suggests that zinc helps maintain tight junction integrity.

Heat stress simultaneously activates inflammation and may suppress adaptive immunity [63]. The cytokine–cytokine receptor interaction pathway was significantly enriched in the H group (−log10(*p*.adjust) ≈ 10.29) but not in the H + Zn group, indicating that heat stress triggers a strong pro-inflammatory response that may be inhibited by zinc. Protein tyrosine kinase activity-related GO terms were enriched only in the H group, further supporting the immunomodulatory role of zinc (Figure 4D). In *Gymnocypris eckloni*, acute heat stress caused intestinal damage, increased plasma IL-1β and TNF-α, and down-regulated adaptive-immunity-related genes (e.g., *mica* and the *hla* family) [63], revealing a dual effect of thermal stress on the immune system. In ayu, a high water temperature induced excessive inflammation and thymic degeneration [64]. In black cusk-eel, heat stress up-regulated pro-inflammatory cytokines such as *tnfa* and *cxcl8* [65]. Notably, the heat-induced transcriptomic reprogramming is not simply an up-regulation of metabolism; in Atlantic salmon, metabolic genes were largely repressed while stress response pathways such as heat shock, ER stress, apoptosis and immune defense were activated [9], reflecting a reallocation of energy resources toward stress responses.

Furthermore, multiple cell death pathways were activated under heat stress. Mitophagy (6.71), autophagy-animal (6.54), necroptosis (2.89) and ferroptosis (2.15) were significantly enriched in the H group but markedly decreased in the H + Zn group (0.11–1.26), suggesting that zinc correlates with attenuated transcriptional activation of the cell death pathways under heat stress (Table S6). In *Huso dauricus*, chronic heat stress induced ferroptosis-mediated lipid metabolism disorder and tissue injury [66]. In pufferfish, high temperature induced apoptosis via the p53-Bax pathway and caspase-dependent cascades [67]. Autophagy and mitophagy are known protective mechanisms in fish under thermal stress, helping to clear damaged mitochondria [68]. The peroxisome (2.00 → 0.85) and glutathione metabolism (0.28 → 1.17) pathways changed between the two groups, reflecting a potential modulatory role of zinc in the transcriptional regulation of redox-related pathways under heat stress [69]. The p53 signaling (3.82 → 2.24) and cellular senescence (6.93 → 2.66) pathways also showed alleviation by zinc.

### 4.2. Zinc-Mediated Regulation of Heat Stress Responses

The sublethal Zn concentration (15 μM, ~50% of the 96 h LC_50_) was selected to avoid direct Zn toxicity while allowing evaluation of its potential alleviating effects on heat-induced intestinal damage, and this level is also environmentally relevant in Zn-polluted waters [70]. Compared with the H group, the number of heat-induced DEGs decreased by 43.2% in the H + Zn group. More importantly, gene cluster and WGCNA module analyses revealed that zinc selectively modulates specific functional modules: clusters highly expressed in the H group were enriched in stress pathways such as proteasome and cell cycle, whereas clusters alleviated in the H + Zn group were enriched in adaptation pathways such as lysosome and FoxO signaling. This suggests that zinc acts as a “fine-tuner”, preserving beneficial adaptive responses while suppressing excessive or damaging ones. In the Zn group, only eight DEGs were identified, demonstrating that the regulatory role of zinc at the experimental concentration is strictly stress-dependent, consistent with its function as a cofactor in stress-responsive pathways. This stress-dependent property reflects the concentration-dependent biphasic effect of essential trace elements and supports the view of zinc as a conditional modulator [69,71].

DNA repair pathways (mismatch repair, base excision repair, and nucleotide excision repair) had low −log10(*p*.adjust) values in the H group (0.14–1.30) but were markedly higher in the H + Zn group (2.08–6.34), indicating that DNA repair pathways may be more active under zinc supplementation. The cell cycle pathway was enriched in both groups, but its −log10(*p*.adjust) was much higher in the H + Zn group (18.37) than in the H group (7.29), suggesting that zinc promotes a “repair-first” strategy, allowing cells to repair DNA damage before division. These findings are consistent with studies in zebrafish embryos, where heat stress facilitated DNA repair and protected against subsequent UV damage [72]. Zinc serves as an essential cofactor for DNA repair enzymes, which likely explains the enhanced repair pathway enrichment observed in the H + Zn group.

In the H + Zn group, the GO terms “protein folding” and “heat shock protein binding” were enriched, and the proteasome pathway was enhanced, indicating that zinc may support proper protein folding and promote the clearance of denatured proteins, thereby reducing ER stress and apoptosis. This role in protein homeostasis may represent an additional protective mechanism. In *Cottus gobio*, combined exposure to heat stress and cadmium induced proteasome activity changes [73]; in notothenioid fishes, thermal adaptation involves the ubiquitin–proteasome pathway [74]. In grass carp, heat shock protein and proteasome subunit genes were up-regulated under heat stress [22]. Thus, zinc-mediated enhancement of the proteasome pathway may contribute to maintaining protein homeostasis under thermal stress.

### 4.3. Co-Expression Networks in Dual Stress Adaptation

WGCNA identified the turquoise module as strongly positively correlated with the high-temperature trait (*r* = 0.93, *p* = 8.6 × 10^−6^). Its 3991 shared genes were enriched in cytoskeleton, focal adhesion and MAPK-FoxO signaling pathways, forming an “emergency defense module” that prioritizes barrier maintenance. The rapid activation of this module may represent an immediate protective response of the intestine to heat stress. Similar WGCNA approaches have revealed heat-responsive modules in turbot (*Scophthalmus maximus*) [20]. The brown module (*r* = 0.70) and grey module (*r* = 0.71) were significantly positively correlated with the H + Zn trait and were enriched in DNA repair, cell cycle and proteasome pathways, forming a “repair adaptation module”. Based on the discussion in Section 4.2, we speculate that instead of simply suppressing the heat-induced response, zinc adds this new functional layer, enabling a transition from passive stress resistance to active repair. This module differentiation reveals a hierarchical regulatory logic in the carp intestine under dual stress.

Although the present study did not directly analyze the gut microbiota, heat-induced disruption of tight junctions and activation of inflammatory pathways create a microenvironment favorable to dysbiosis. In *Gymnocypris eckloni*, heat stress caused intestinal damage, increased permeability, and shifted the microbiota from *Aeromonas*-dominated to *Shewanella*-dominated [63]. In rainbow trout, heat stress significantly altered the mucosal microbiota, with increased abundance of *Acinetobacter* and *Enterobacteriaceae* [58]. In the present study, zinc reduced tight junction enrichment (Figure 6F) and suppressed inflammation, suggesting that zinc may indirectly support a healthy microbiota. Future studies integrating metagenomics are needed to directly verify the regulatory effect of zinc on the gut microbiota.

The limitation of this study is that all analyses were based solely on transcriptomic data. Although RNA-seq results were validated by qPCR, no functional validation (e.g., CRISPR) or physiological measurements (e.g., intestinal permeability, protein levels of tight junctions, and oxidative stress biomarkers) were performed. Thus, the proposed mechanisms remain at the transcriptional level and require experimental verification. Only a single sublethal concentration of waterborne zinc (15 μM) was tested; a concentration gradient is needed to determine dose-dependent effects and the optimal concentration. The potential effects on gut microbiota were inferred only from transcriptomic evidence of barrier integrity and immune modulation, as direct microbial analysis (e.g., 16S rRNA sequencing) was not conducted. Therefore, the conclusion that zinc may indirectly support a healthy microbiota remains hypothetical. Finally, transcriptomics does not capture post-transcriptional, translational or metabolic regulation; integration of multi-omics approaches (e.g., proteomics and metabolomics) is needed for a more comprehensive understanding.

## 5. Conclusions

This study took common carp as the research object, adopted a 2 × 2 factorial experimental design, and combined technologies such as Illumina high-throughput RNA sequencing, differential expression gene analysis, GO/KEGG functional enrichment, and weighted gene co-expression network analysis (WGCNA) to systematically decipher the transcriptomic response characteristics of carp intestinal tissues under single and combined treatments of heat stress and sublethal waterborne zinc exposure, as well as to clarify the molecular regulatory mechanism by which zinc suggests a potential modulatory role in the intestinal transcriptional response to heat stress in carp. The results confirmed that the transcriptome sequencing data are reliable with good biological repeatability, which can support subsequent analyses. Heat stress is the core environmental driver triggering global reprogramming of the gene expression network in the common carp intestine, primarily by disrupting the intestinal barrier (including tight junction disruption, focal adhesion and cytoskeleton remodeling), activating inflammatory responses (e.g., cytokine–cytokine receptor interaction), and inducing multiple cell death pathways (including autophagy, necroptosis and ferroptosis), leading to the generation of a large number of DEGs. Zinc at a sublethal concentration of 15 μM reduced the number of heat-induced DEGs by 43.2% and correlated with modulated transcription of genes related to intestinal cell damage and protein homeostasis under heat stress, which was linked to transcriptional regulation of the cell cycle, DNA mismatch repair, and the ubiquitin–proteasome pathway. WGCNA identified core modules responsive to high temperature and zinc-specific mitigation modules, revealing two distinct stress adaptation strategies: “emergency defense” and “repair and adaptation”. This study enriches the theoretical system of environmental stress adaptation in teleosts, provides candidate targets for the molecular breeding of heat-tolerant carp varieties, and provides a theoretical basis for the scientific application of zinc in the healthy culture of common carp during high-temperature seasons.

## Figures and Tables

**Table 1:** Summary of Illumina RNA-Seq data.

Sample	Raw Reads Number	Clean Reads Number	Raw Bases (bp)	Clean Bases (bp)	Q20 (%)	Q30 (%)	GC Content
C-1	50,416,436	48,747,934	7,612,881,836	7,307,488,228	97.99	94.71	43.34
C-2	44,808,828	43,837,856	6,766,133,028	6,591,368,894	98.4	95.95	41.81
C-3	48,566,508	47,505,852	7,333,542,708	7,135,610,336	98.36	95.84	42.37
H-1	40,313,002	39,549,320	6,087,263,302	5,954,748,335	98.57	96.14	45.01
H-2	40,843,422	39,786,706	6,167,356,722	5,984,321,501	98.06	94.8	45.43
H-3	42,219,344	41,610,548	6,375,120,944	6,268,797,214	98.93	97.08	44.6
Zn-1	44,534,930	43,414,728	6,724,774,430	6,523,201,299	98.41	95.93	42.95
Zn-2	42,263,206	41,357,444	6,381,744,106	6,219,696,666	98.41	95.94	42.24
Zn-3	45,623,520	43,889,000	6,889,151,520	6,590,457,092	97.92	94.39	44.54
H + Zn-1	56,086,074	55,098,142	8,468,997,174	8,292,685,814	98.63	96.4	43.46
H + Zn-2	39,884,110	39,180,344	6,022,500,610	5,897,198,158	98.65	96.44	43.51
H + Zn-3	37,420,854	36,728,126	5,650,548,954	5,527,373,339	98.57	96.2	44.34
Total	532,980,234	520,706,000	80,480,015,334	78,292,946,876	-	-	-

Notes: Q20: the correct recognition rate of bases is 99%; Q30: the correct recognition rate of bases is 99.9%; GC content: the total number of bases G and C as a percentage of the number of four bases.

**Table 2:** Results of comparison between sample and reference genome.

Sample	Total_Mapped (%)	Multiple_Mapped (%)	Uniquely_Mapped (%)
C-1	40,153,703 (82.37%)	3,000,918 (7.47%)	37,152,785 (92.53%)
C-2	35,943,046 (81.99%)	2,879,506 (8.01%)	33,063,540 (91.99%)
C-3	39,855,612 (83.90%)	2,843,066 (7.13%)	37,012,546 (92.87%)
H-1	31,945,500 (80.77%)	4,018,853 (12.58%)	27,926,647 (87.42%)
H-2	31,588,209 (79.39%)	4,002,650 (12.67%)	27,585,559 (87.33%)
H-3	33,356,278 (80.16%)	4,184,970 (12.55%)	29,171,308 (87.45%)
Zn-1	36,229,116 (83.45%)	2,669,569 (7.37%)	33,559,547 (92.63%)
Zn-2	33,947,231 (82.08%)	2,726,464 (8.03%)	31,220,767 (91.97%)
Zn-3	36,100,302 (82.25%)	2,851,792 (7.90%)	33,248,510 (92.10%)
H + Zn-1	44,785,770 (81.28%)	4,741,039 (10.59%)	40,044,731 (89.41%)
H + Zn-2	31,983,879 (81.63%)	3,416,982 (10.68%)	28,566,897 (89.32%)
H + Zn-3	29,523,801 (80.38%)	3,790,732 (12.84%)	25,733,069 (87.16%)

Notes: Unique Mapped: the number of clean reads that uniquely mapped onto the reference genome; Multiple Mapped: the number of clean reads that mapped onto multiple loci of the reference genome; Total Mapped: the number of clean reads that mapped onto the reference genome.

## Data Availability

Illumina sequencing raw reads data and transcripts sequences have been uploaded to the NCBI SRA database; the item number is PRJNA1432439.

## Supplementary Materials

The following supporting information can be downloaded at https://www.mdpi.com/article/10.3390/ani16091334/s1, Figure S1: Volcano plots of differentially expressed genes (DEGs) in the intestinal tissue of common carp (*Cyprinus carpio*) under different stress conditions; Figure S2: Gene co-expression clustering of the intestinal transcriptome in common carp under different stress conditions; Figure S3: Gene Ontology (GO) enrichment analysis of DEGs in common carp intestinal tissue across six pairwise stress comparisons; Figure S4: Venn diagram of the overlap between genes identified by WGCNA and DEGs under zinc-added treatment; Figure S5: GO enrichment analysis of differentially expressed genes in common carp intestinal tissue under heat stress (C vs. H) and heat and zinc-added combined stress (C vs. H + Zn); Figure S6: Gene-pathway interaction networks of DEGs in common carp intestinal tissue under C vs. H and C vs. H + Zn; Figure S7: Schematic diagram of the experimental design. Table S1: Specific primers for nine verified genes were used for qRT-PCR; Table S2: FPKM values and DEG analysis results of common carp intestinal transcriptome under different treatments. Table S3: DEGs were identified across the six comparison groups; Table S4: GO enrichment analysis of DEGs in each pairwise comparison; Table S5: Summary of WGCNA module statistics and intramodular connectivity (kIM) for each gene; Table S6: KEGG pathway enrichment analysis of genes commonly identified by DEG analysis and WGCNA in C vs. H and C vs. H + Zn comparisons; Table S7: GO enrichment analysis of genes commonly identified by DEG analysis and WGCNA in C vs. H and C vs. H + Zn comparisons.
