# Supplementary material for: Transcriptomic Response and Molecular Adaptation Mechanisms of Common Carp (Cyprinus carpio) Intestine Under Dual Stress of High Temperature and Zinc
Source: Animals (Basel). 2026 Apr 27;16(9):1334. doi: 10.3390/ani16091334 (PMC13162933; doi:10.3390/ani16091334)
Supplement: Supplementary file 1 [file animals-16-01334-s001.zip › Figure S7.pdf]

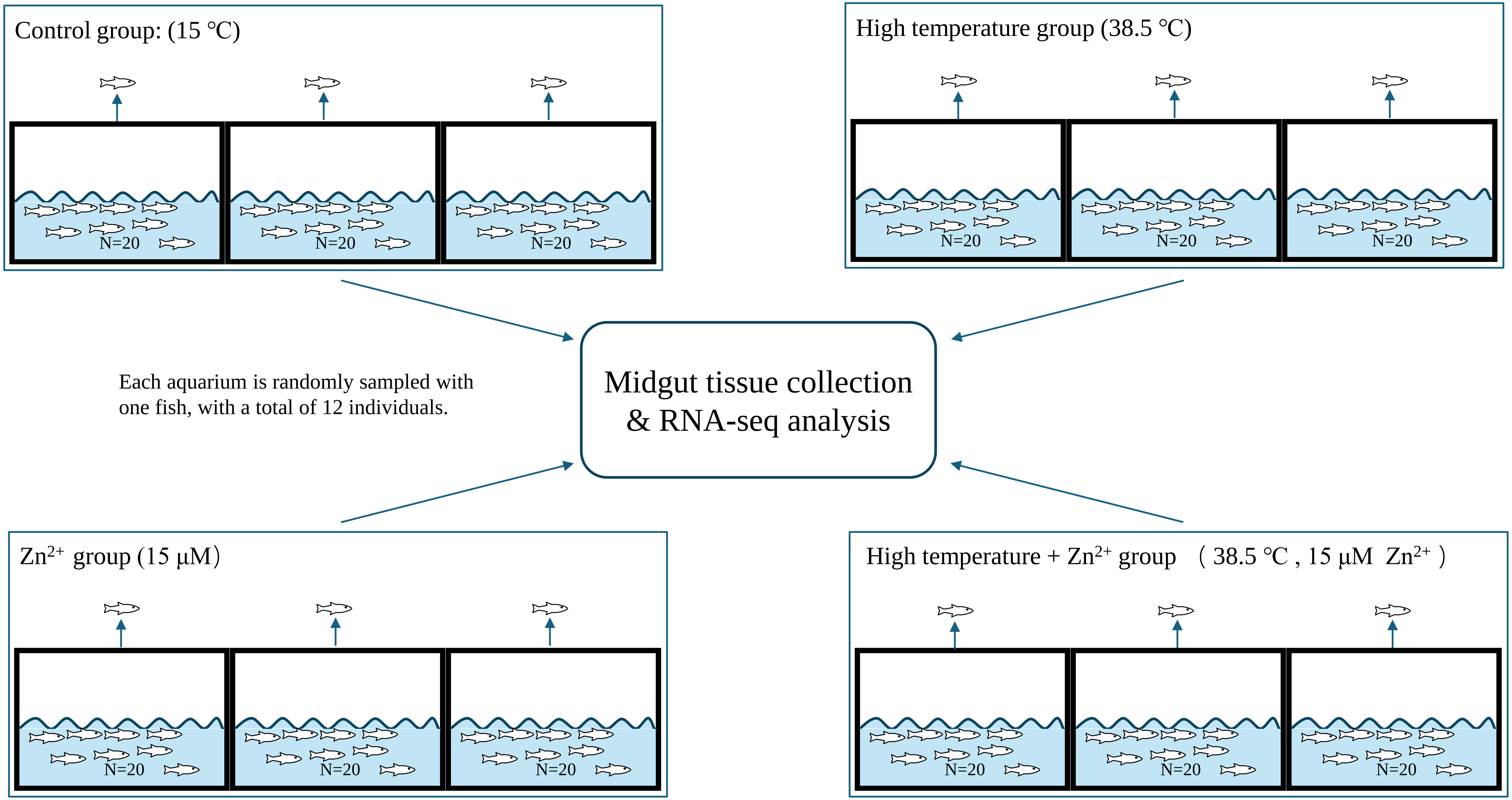

Note: Prior to the formal experiment, the water temperature of the high-temperature group and high-temperature + Zn combined group was gradually increased to 38.5 °C at 4 °C / h. After 24 h of temperature stabilization, the formal experiment was initiated, with a treatment cycle of 21 d.
