# Supplementary figures and images for: Transcriptomic Response and Molecular Adaptation Mechanisms of Common Carp (Cyprinus carpio) Intestine Under Dual Stress of High Temperature and Zinc
Source: Animals (Basel). 2026 Apr 27;16(9):1334. doi: 10.3390/ani16091334 (PMC13162933; doi:10.3390/ani16091334)

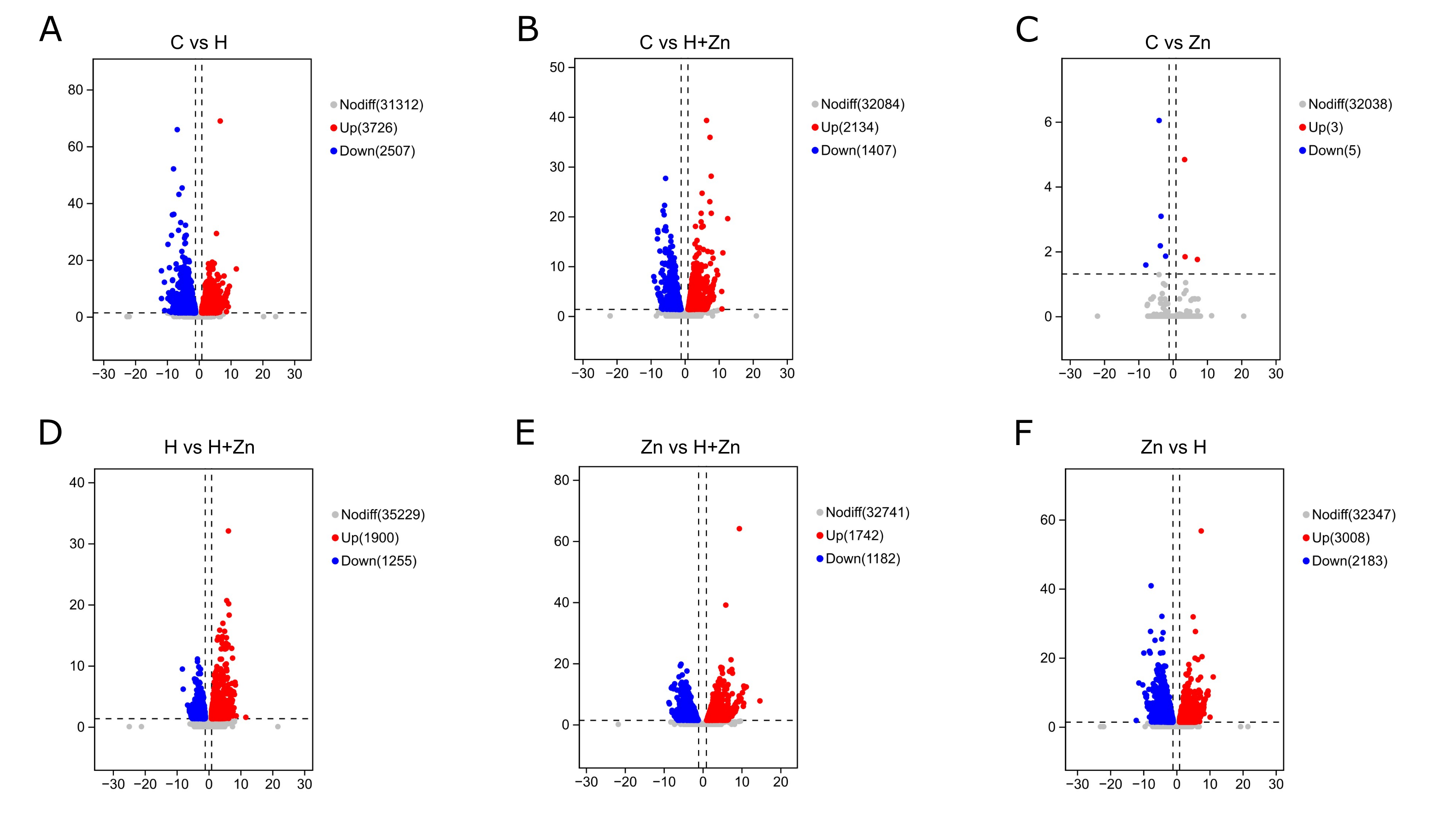

Supplement: Supplementary file 1 [file animals-16-01334-s001.zip › Figure S1.tiff]

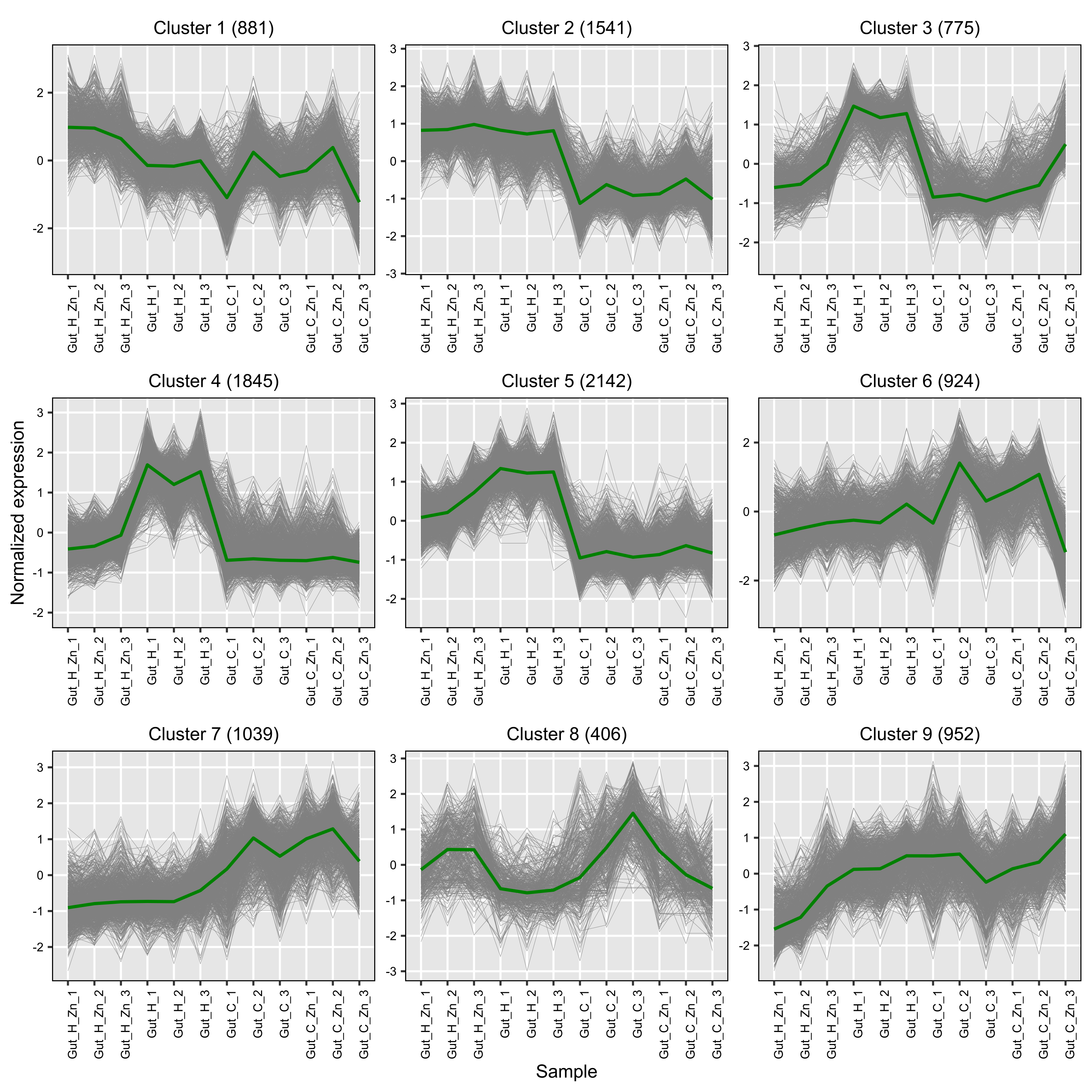

Supplement: Supplementary file 1 [file animals-16-01334-s001.zip › Figure S2.tiff]

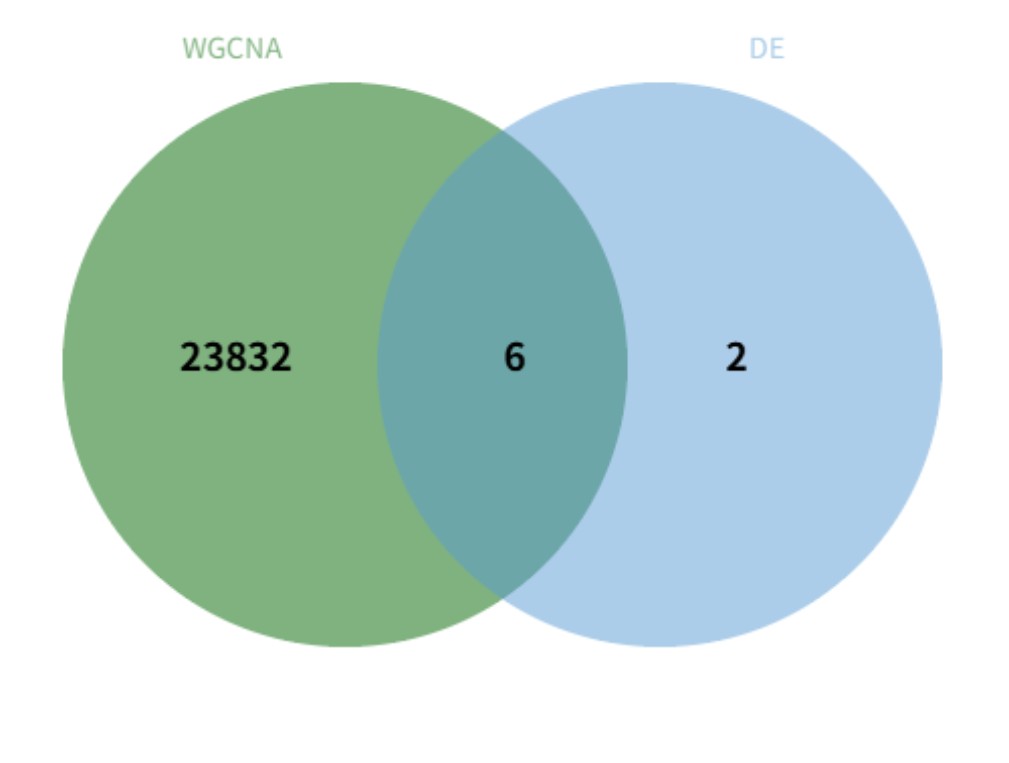

Supplement: Supplementary file 1 [file animals-16-01334-s001.zip › Figure S4.jpg]

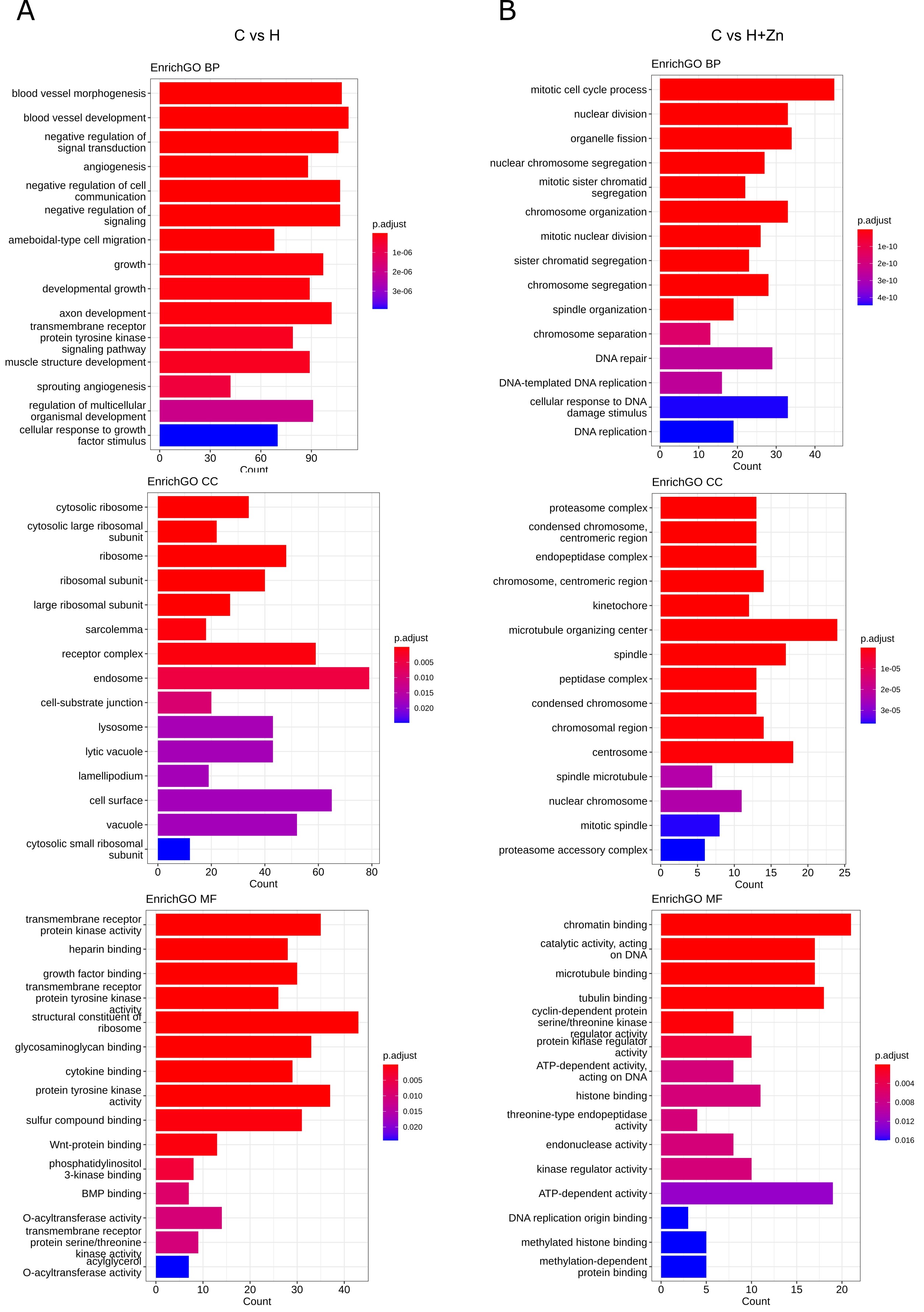

Supplement: Supplementary file 1 [file animals-16-01334-s001.zip › Figure S5.jpg]

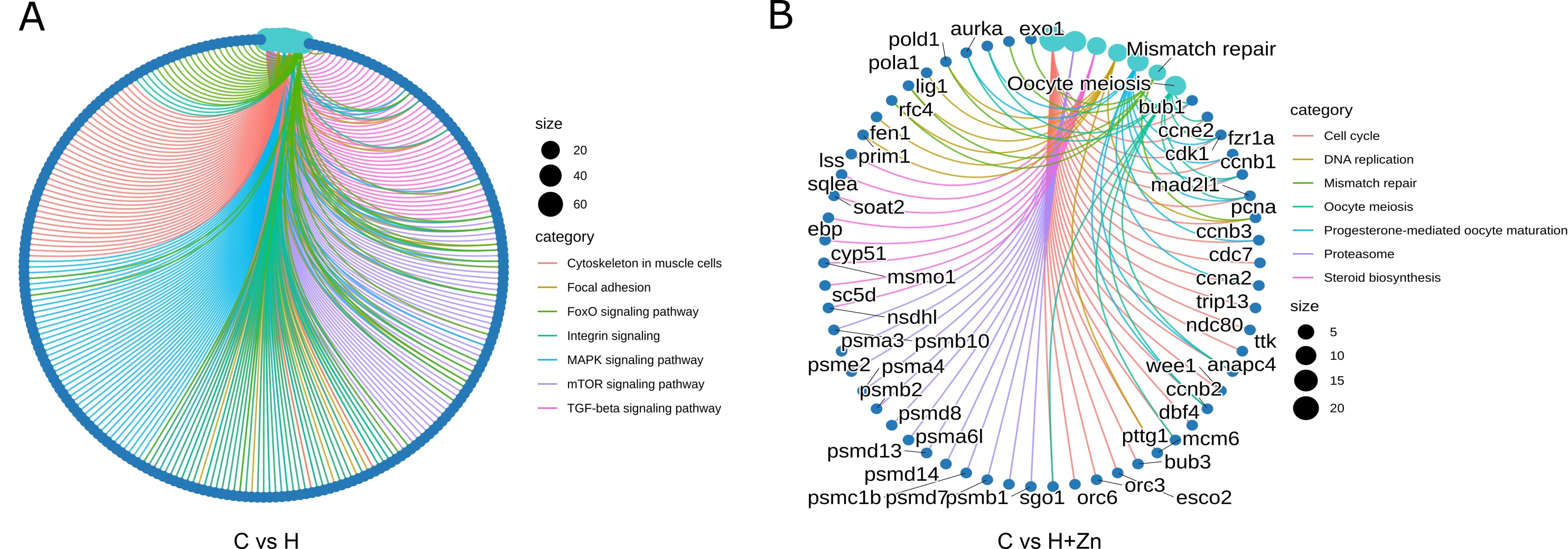

Supplement: Supplementary file 1 [file animals-16-01334-s001.zip › Figure S6.jpg]
